# The β-oxidation pathway is downregulated during diapause termination in *Calanus* copepods

**DOI:** 10.1038/s41598-019-53032-5

**Published:** 2019-11-13

**Authors:** Elise Skottene, Ann M. Tarrant, Anders J. Olsen, Dag Altin, Mari-Ann Østensen, Bjørn Henrik Hansen, Marvin Choquet, Bjørn M. Jenssen, Rolf Erik Olsen

**Affiliations:** 1 Department of Biology, NTNU, Trondheim, Norway; 2 Woods Hole Oceanographic Institution, Woods Hole, Massachusetts, USA; 3 BioTrix, Trondheim, Norway; 4 SINTEF Ocean AS, Environment and New Resources, Trondheim, Norway; 5 Faculty of Biosciences and Aquaculture, Nord University, Bodø, Norway

**Keywords:** Developmental biology, Transcriptomics, Fat metabolism

## Abstract

*Calanus* copepods are keystone species in marine ecosystems, mainly due to their high lipid content, which is a nutritious food source for e.g. juvenile fish. Accumulated lipids are catabolized to meet energy requirements during dormancy (diapause), which occurs during the last copepodite stage (C5). The current knowledge of lipid degradation pathways during diapause termination is limited. We characterized changes in lipid fullness and generated transcriptional profiles in C5s during termination of diapause and progression towards adulthood. Lipid fullness of C5s declined linearly during developmental progression, but more β-oxidation genes were upregulated in early C5s compared to late C5s and adults. We identified four possible master regulators of energy metabolism, which all were generally upregulated in early C5s, compared to late C5s and adults. We discovered that one of two enzymes in the carnitine shuttle is absent from the calanoid copepod lineage. Based on the geographical location of the sampling site, the field-samples were initially presumed to consist of *C. finmarchicus*. However, the identification of *C. glacialis* in some samples underlines the need for performing molecular analyses to reliably identify *Calanus* species. Our findings contributes to a better understanding of molecular events occurring during diapause and diapause termination in calanoid copepods.

## Introduction

Marine zooplankton, such as copepods, form a critical trophic link between phytoplankton production and predators from higher trophic levels in marine ecosystems^1,2^. The crustaceans *Calanus finmarchicus* and *Calanus glacialis* are calanoid copepod species distributed mainly in boreal, sub-arctic and arctic water masses, that comprise up to 90% of the total mesozooplanktonic biomass in the North Atlantic Ocean^3–5^. During their life cycle, both *C. finmarchicus* and *C. glacialis* develop through six naupliar (larval) and five copepodite (juvenile) stages, before molting into the terminal reproductive adult stage. During the last copepodite stage (C5), most of the individuals enter into a facultative dormant phase, termed “diapause.” This phase is preceded by accumulation of energy stores as wax esters (esters of a fatty acid and a fatty alcohol), which are stored in a specialized organ called the lipid sac. At onset of diapause, the copepods undergo vertical migration to deeper and colder water layers, where the diapausing copepods will remain for several months without feeding. The wax esters are catabolized during dormancy and they provide energy for locomotion, molting, gonad maturation and reproduction after emergence from diapause^6–8^. The importance of *C. finmarchicus* and *C. glacialis* in marine food webs relies on the copepods’ ability to accumulate and store large amount of energy-rich lipids, converted from their primarily phytoplankton diet^9^. This conversion allows an abundant food source to become available to higher trophic level fishes including juvenile Atlantic cod (*Gadus morhua)*^10^ and herring (*Clupea harengus*)^1^ when the copepods terminate diapause and ascend to surface waters. To understand marine ecosystem dynamics and the complex interactions between trophic levels, more knowledge is needed about the basic physiology of these ecologically important species.

Several important life history traits in these calanoid copepod species depend on precise regulation of catabolism of stored lipids to provide energy for processes like oogenesis, molting^7,11^ and preceeding this, presumably diapause termination^12,13^. Current knowledge suggests that metabolism decreases drastically during the first phase of diapause (“the initiation phase”)^12,14^. During “the maintenance phase”, metabolic rate remains low and constant, and the copepods therefore only slowly deplete their lipid stores^14^. Physiological development and gonadogenesis may be initiated within the maintenance phase^15^, before unknown processes result in an increased sensitivity to diapause terminating conditions, which results in awakening^14,15^. The current understanding of the process of this “termination phase” is very poor. Termination eventually leads to the resumption of development and the copepods then migrate to upper water layers to molt into adults and reproduce^14,15^. The molecular basis of lipid biosynthesis as a part of diapause preparations has previously been examined, primarily in *C. finmarchicus*^16,17^. Recently, transcriptomic changes during emergence from diapause have been investigated in the Pacific calanoid copepod *Neocalanus flemingeri*^18^. Beyond these studies, genomic resources for copepods are limited^16,19^, and the molecular basis of pathways for lipid degradation and other catabolic processes remains poorly understood in both *C. finmarchicus* and *C. glacialis*, particularly in the context of diapause termination.

Lipid degradation processes are broadly conserved across species, from archaea^20^ to mammals^21^. In marine zooplankton species that preferentially store wax esters as their dominant storage lipids, the wax esters are first hydrolyzed into fatty acids and fatty alcohols^22^. The fatty acids can then enter the β-oxidation pathway, which involves transportation into mitochondria or peroxisomes, before each hydrocarbon chain is shortened by one fatty acyl group at a time, ultimately producing molecules of fatty acetyl-CoA, which are used in the citric acid cycle for ATP generation (e.g.^21^). Each reaction in this pathway is catalysed by families of catabolic enzymes, such as transferases, ligases, dehydrogenases, oxidases and hydrolases. Regulation and activities of catabolic enzymes are finely tuned in order to optimally preserve cell integrity^23^. Certain genes that are involved in directing this regulation are called “master regulators”^24^. Several master regulators of energy metabolism have been characterized within invertebrate model species, such as tumor protein p63 (TAp63)^25^, sterol regulatory element-binding protein (SREBP)^26^ and the nuclear receptors E-75^27^ and hepatocyte nuclear factor 4 (HNF4)^28,29^. None of these genes have been investigated in *C. finmarchicus* or *C. glacialis*. Due to their highly conserved roles in other invertebrates, they may also be important in regulating energetic metabolism in these copepod species. The genes ferritin, heat shock protein 22 (hsp22) and torso-like protein have been specifically linked to developmental progression and/or diapause in *C. finmarchicus*^17,30,31^. Analyses integrating such biomarkers of energetic metabolism, development and diapause may contribute to elucidating the regulation of lipid metabolism occurring during the termination of diapause in marine copepods.

The primary goal of this study was to use transcriptional profiling to characterize changes in energy metabolism in copepods during the termination of diapause, and during development from the early C5 stage through to the final molt into adulthood. Specifically, we focused on expression of genes involved in β-oxidation of lipids, and genes that have previously been related to diapause or development in calanoid copepods. In order to evaluate changes in gene expression over time, we collected copepods in diapause from the Trondheimsfjord, Norway, and transferred them to laboratory facilities on shore. Here, the copeods were kept during diapause termination and subsequent molting into adults (approximately one month after collection). These copepods, henceforth refered to as the “experimental” copepods, were sampled for RNA sequencing and lipid fullness ratio analyses at four time points during the experimental period (Table 1, see methods). The experimental group was compared to a group of copepods that were in an early diapause state (the reference group, sampled on the research vessel immediately after collection). Through this approach, we attempted to reveal the molecular pathways that underlie physiological processes occurring during diapause maintenance and termination in keystone marine copepods.

**Table 1:** Sampling scheme of the experiment.

Sampling event	Day no.	Stage	N per sample
Early diapause group	Reference group	C5	10
Sorting and experiment start, “zero” sampling	0	C5	3
1^st^ sampling	5	C5	3
2^nd^ sampling	13	C5 & Adults	3 & 3
3^rd^ sampling	20	C5 & Adults	3 & 3

## Results

### The presence of *C. glacialis*

After the completion of sampling and RNA sequencing within the present study, the occurrence of the “true Arctic shelf species” *Calanus glacialis*^3^ was documented in the Trondheimsfjord through molecular analyses^32,33^. Molecular-based species identification analyses showed that there were individuals of *C. glacialis* present together with *C. finmarchicus* in several samples. Both species were present in two samples from the reference group (the early diapause copepodites sampled on the research vessel, see methods), in two C5 samples from day 0, in one adult sample from day 13 and in one adult sample from day 20 (see Supplementary Table S1). The C5 sample from day 20 contained 100% of *C. glacialis*. In total, seven samples contained 100% *C. finmarchicus*, one contained 100% *C. glacialis* and six contained a mix of both species (Table S1). As the species identification analyses was performed on RNA aliquots of pooled individuals, the percentage of each species in the “mix” samples is undeterminable.

### Improved annotation of the *C. finmarchicus* transcriptome

Inspection of available *C. finmarchicus* transcriptomes^17,34^ (see methods) revealed that the automated annotation pipelines used did not comprehensively identify the genes corresponding to enzymes in the β-oxidation pathway. Manual annotation of these genes, using the amino acid sequences of all enzymes in the β-oxidation pathway in *D. pulex* available in the Kyoto Encyclopaedia of Genes and Genomes database (KEGG) as queries when blasting in the reference transcriptome, resulted in successful identification of all these enzymes in *C. finmarchicus*, with the exception of two enzymes (see Supplementary Table S2).

**Figure 1:**
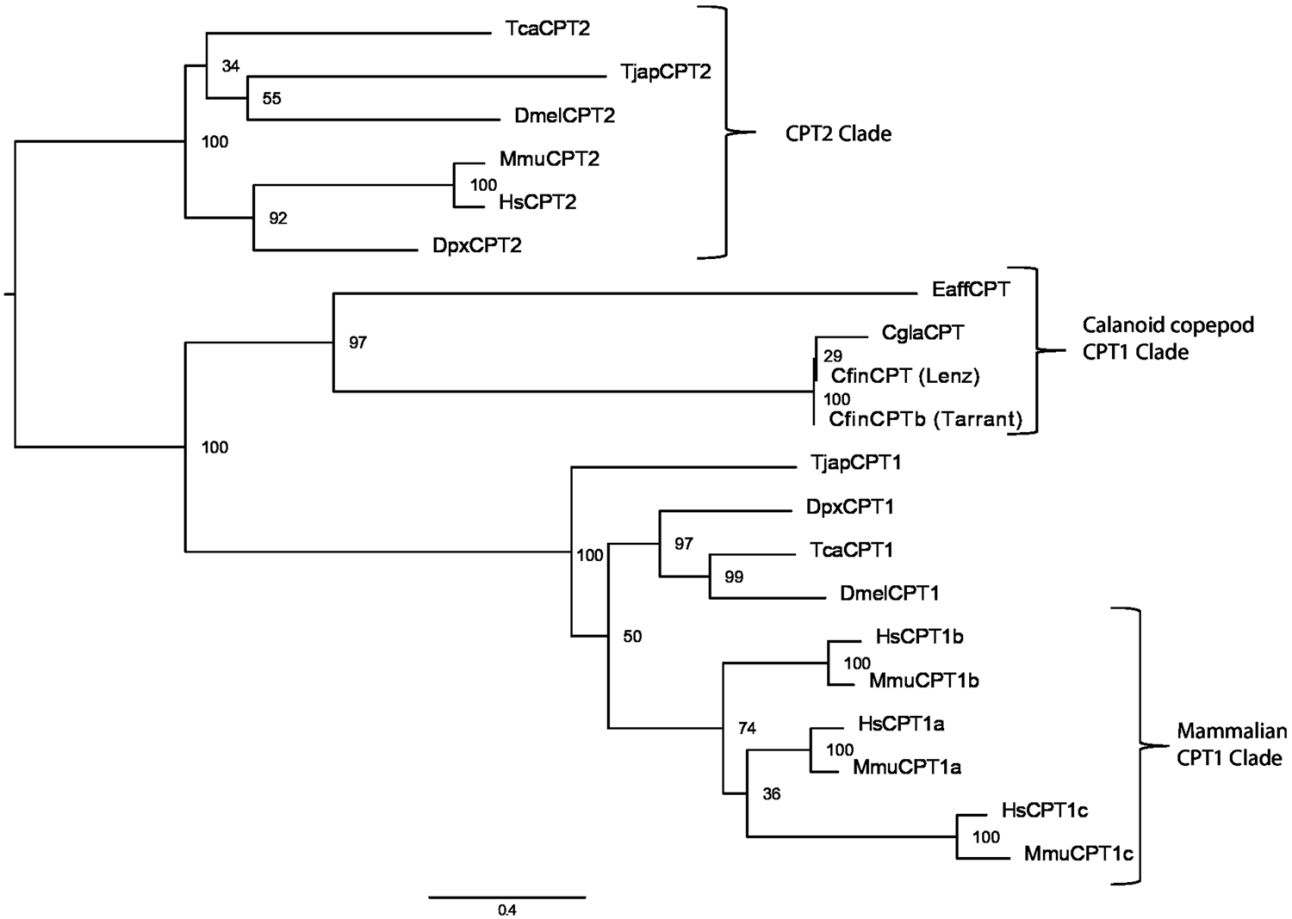
Maximum likelihood analysis of carnitine O-palmitoyltransferase (CPT) genes from calanoid copepods (*Calanus finmarchicus*, Cfin*; C. glacialis*, Cgla; *Eurytemora affinis*, Eaff) other arthropods (*Tigriopus japonicas*, Tjap; *Daphnia pulex*, Dpx; *Tibolium castaneum*, Tca; *Drosophila melanogaster*, Dmel) and mammals (*Homo sapiens*, Hs; *Mus musculus*, Mmu). Numbers on nodes represent the percentage of 1000 bootstrap replicates supporting the grouping. Scale indicates substitutions per site (i.e. bar length of 0.4 shown). See also Supplementary Table S8 for accession numbers.

Of the two enzymes in the carnitine shuttle, which transports fatty acids into mitochondria (carnitine O-palmitoyltransferase, CPT, EC 2.3.1.21), only one form (CPT1) was present in the *C. finmarchicus* reference transcriptome. Similar searches of a second *C. finmarchicus* transcriptome, two transcriptomes for *C. glacialis* and a *Eurytemora affinis* transcriptome (see methods for NCBI accession numbers), revealed only a single CPT homolog in these calanoid copepod species. In contrast, both CPT1-like and CPT2-like transcripts were identified through searches in the harpacticoid copepod *Tigriopus japonicus*. A maximum likelihood analysis confirmed that all copepod species contained CPT1 orthologs, but CPT2 was only present in *T. japonicus* (Fig. 1). The analysis correctly recovered the CPT1 and CPT2 clades with 100% bootstrap support. Within the CPT1 clade, the mammalian (*Homo sapiens, Mus musculus*) sequences fell into a moderately supported (74%) monophyletic clade, with multiple forms in each species due to diversification early in the deuterostome lineage. The arthropod sequences (*C. finmarchicus, C. glacialis, E. affinis, T. japonicus, D. pulex, Tibolium castaneum*, and *Drosophila melanogaster*) did not form a monophyletic group, and the calanoid copepod sequences were positioned on well-supported clade (97%) with relatively long branches.

The *D. pulex* genes encoding butyryl-CoA dehydrogenase (BCD, EC 1.3.8.1) and short/branched chain acyl-CoA dehydrogenase (EC 1.3.99.12) had the same top hit in the *C. finmarchicus* transcriptome (comp266079_c0_seq1). The gene was annotated as a mitochondrial short chain specific acyl-CoA dehydrogenase (see Supplementary Table S2), most likely signifying EC 1.3.99.12, which means that EC 1.3.8.1 (BCD) was not identified in the transcriptome.

### Lipid fullness ratio

**Figure 2:**
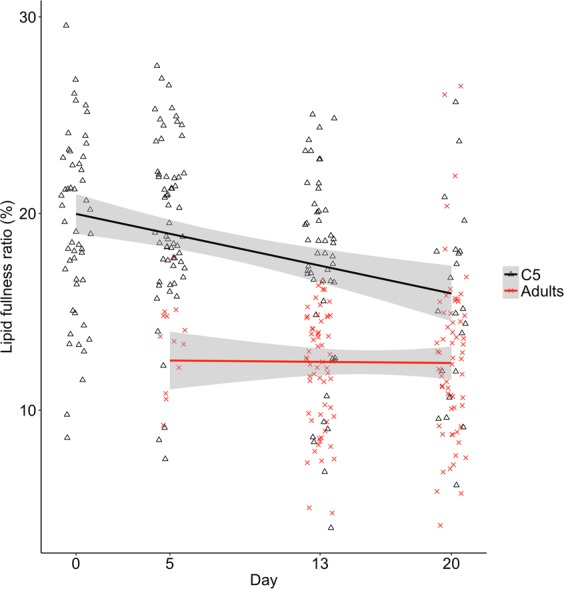
Lipid fullness ratio of experimental *Calanus* spp. C5s (n = 185) and adults (n = 136, N = 321). There was a significant decline in lipid fullness ratio with time in C5s (estimate ± s.e.m. = −0.20% ± 0.09 per day, P = 0.029), and no decline in adults (estimate ± s.e.m. = −0.01% ± 0.08 per day, P = 0.91). ANCOVA: F_3,315_ = 60.22, R^2^ = 0.37. Regression lines are shown with confidence intervals.

Mean lipid fullness ratio, measured as (lipid sac volume/prosome volume) * 100, was highest in the reference group (early diapause C5s), followed by the experimental C5s sampled on days 0, 5, 13 and 20 (Table 2). The lipid fullness ratio was lower in adults than in C5s at all time points. ANCOVA results showed that the lipid fullness ratio declined with time (days since intitation of experiment) in the experimental C5s, but not in the adults (not present on day 0, *P* = 0.029, Fig. 2). The C5s (*n* = 185) showed a decline in lipid fullness ratio of 0.20% ± 0.09 (s.e.m.) per day, while the adults (*n* = 136) had a stable lipid fullness ratio over time (estimate ± s.e.m. = −0.01% ± 0.08 per day).

**Table 2:** Lipid fullness ratio (means and s.d.) in *Calanus* spp. C5s and adults at all sampling times. Lipid fullness ratios were calculated as percentage of lipid sac volume relative to prosome volume (see methods).

Stage	Sampling time	Mean (%)	s.d.	n
C5	Reference group	22.32	9.21	55
	Day 0	19.61	4.78	52
	Day 5	19.6	4.22	61
	Day 13	17.42	4.87	49
	Day 20	16.23	6.23	23
Adults	Day 0	NA	NA	0
	Day 5	13.46	2.29	13
	Day 13	12.02	2.91	60
	Day 20	12.62	4.26	63

### Transcriptomic temporal variation

**Figure 3:**
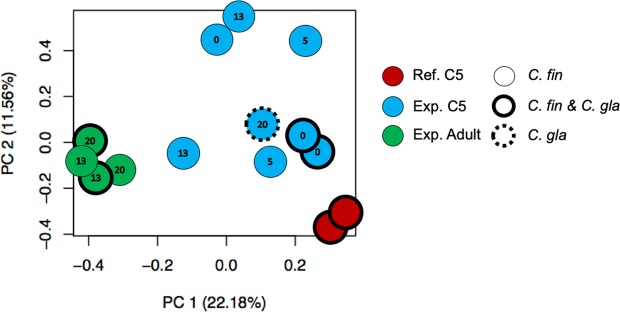
Principal Component Analysis (PCA) plot of gene expression patterns in all *Calanus* spp. samples. The numbers in the symbols indicate the sampling day, i.e. 0, 5, 13 or 20. The borders surrounding the symbols indicate species, i.e. *C. finmarchicus* (*C.fin*), *C. glacialis* (*C.gla*) or a mix of the two species. C5s in the reference (Ref) group (red, in early diapause) clustered to the far right, and experimental (Exp) adults (green) to the far left. The experimental C5s (blue) were positioned somewhat variably between the reference group and adults, which may suggest some asynchronization in development and diapause phase.

A principal components analysis (PCA) of all genes in all samples (C5s and adults) showed a clear separation along principal component 1 (PC1, 22.18% variation explained) between the reference group (early diapause C5s, see methods) and adults on day 13 and 20 (Fig. 3). In contrast, the experimental C5s scattered along PC1 between the reference group to the right and the adults to the left. These early and late C5s appear somewhat more differentiated from the reference group and had higher PC2 values (PC2: 11.56% variation explained).

**Figure 4:**
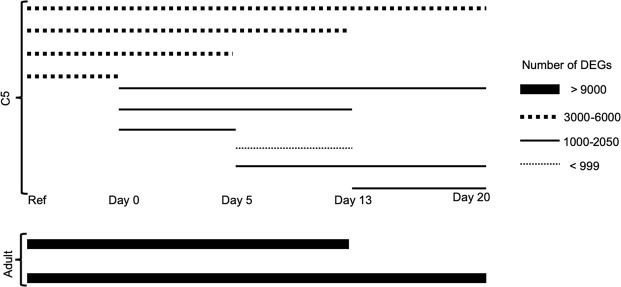
Illustration of the number of differentially expressed genes (DEGs) between comparisons, analyzed by GLMs. All DEGs are listed in Supplementary Table S3. The start and end of each line indicates which comparison the line refers to. The thicker the line, the more DEGs between the compared groups, e.g. >9000 DEGs between the reference group and adults sampled on days 13 and 20. The thinner line, the fewer DEGs, e.g. <999 DEGs between experimental C5s sampled on days 5 and 13.

Differentially expressed genes (DEGs) between all compared sampling days were analyzed with generalized linear models (GLM, see methods). The number of DEGs (P > 0.05) ranged from 911 to 10580 (Fig. 4, number of all DEGs listed in Supplementary Table S3). The number of DEGs between the reference group and the adults sampled on days 13 and 20 was higher than 9000 (Fig. 4). There was also a large number of DEGs between the reference group and the experimental C5s (3000-6000 DEGs). Comparisons within the experimental C5s (days 0-20) had generally lower numbers of DEGs (900-2050 DEGs), while comparisons between experimental C5s and adults were around 6500 DEGs (Supplementary Table S3).

### Gene ontology (GO) enrichment analysis

To identify processes that are potentially associated with diapause termination, a comparative GO enrichment analysis was performed based on DEGs between the reference group and all experimental C5s combined (days 0, 5, 13 and 20). Among upregulated genes in the reference group, 646 GO terms were significantly enriched (P < 0.05), while 497 GO terms were enriched among the upregulated genes in the experimental C5s. The GO terms with the most statistically significant enrichment (lowest *P*-values, i.e. least likely to be occur by chance. See Supplementary Fig. S1) in the reference group include 14 processes involved in the immune and/or defense system (e.g. GO:0006955: immune response, GO: 0006952: defense response and GO: 0038061: NIK/NF-kappaB signaling), as well as developmental processes of cells, neurons and synapses and endopeptidase activity. In the experimental C5s, the most statistically significant enriched GO terms (Supplementary Fig. S2) include GO:0007283: spermatogenesis and GO:0007281: germ cell development, along with cuticle, chitin and epidermal developmental related processes. Other terms that were upregulated in the experimental C5s were mostly related to cell division and development in general.

### Differential expression of genes of interest

**Figure 5:**
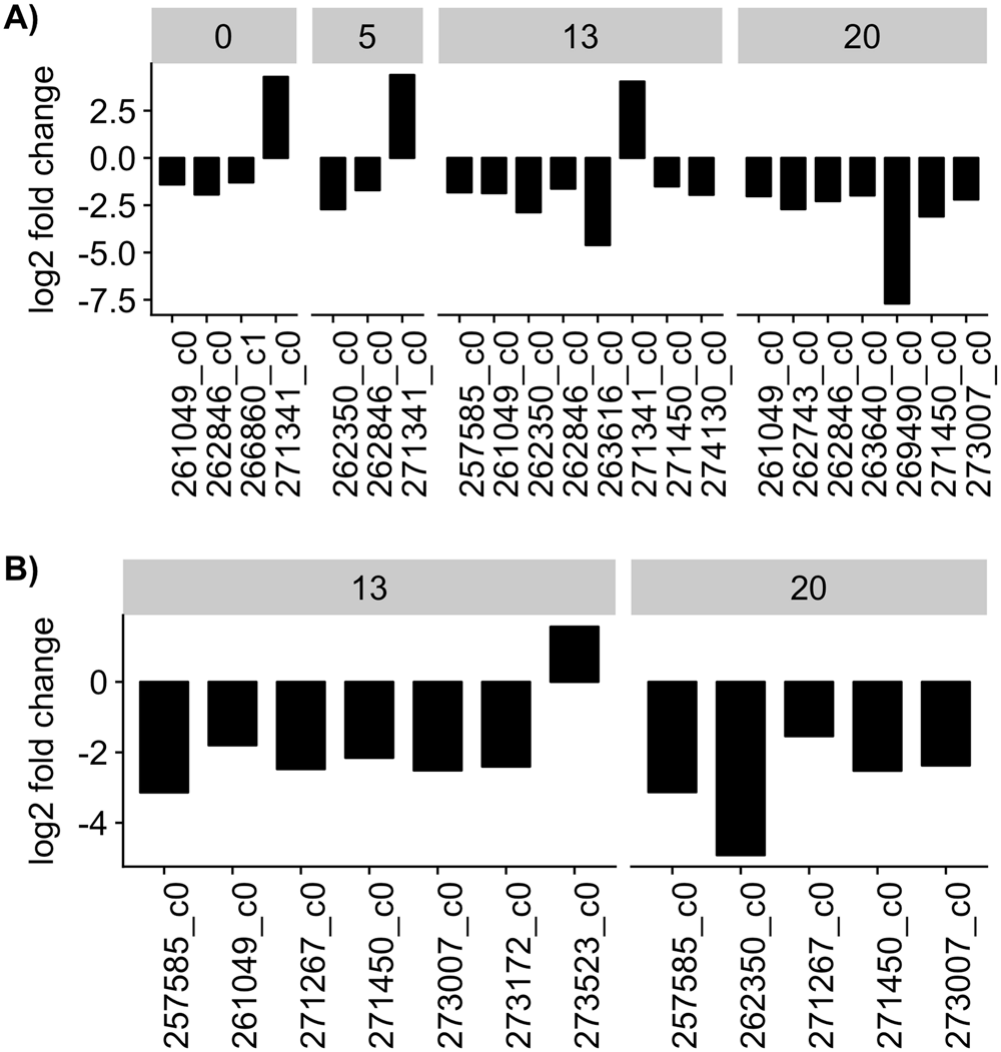
Significantly differentially expressed genes (P < 0.05) in the β-oxidation pathway in experimental C5s and adult *Calanus* spp. compared to the reference group. (**A**) In C5s, more β-oxidation genes were downregulated compared to the reference group on days 13 and 20 (seven genes)  than on days 0 (three genes) and 5 (two genes). (**B**) Adults appeared in sufficient numbers for sampling on day 13. Six and five genes were downregulated in adults on days 13 and 20 compared to the reference group, respectively. Differences in expression is shown as log2 fold change in each group compared to the reference group. For example, a log2 fold change of -2 signifies a 4 fold decrease from the reference group. The bar at the top indicates day of sampling. The X-axis shows the gene ID (see Supplementary Table S4 for annotation and GLM metrics).

The GLM results showed that several genes in the β-oxidation pathway were significantly (P < 0.05) downregulated in the experimental C5s compared to the reference group (Fig. 5A, comprehensive GLM metrics in Supplementary Table S4). Among these, more genes were downregulated in late C5s (days 13 and 20, seven genes, logFC range: −1.5 to −3.1, P < 0.05) than in early C5s (days 0 and 5, three genes, logFC range: −1.3 to −2.7, P < 0.05). One β-oxidation gene was upregulated on days 0, 5 and 13 (logFC: ~4.0, P < 0.05). Several genes were also downregulated in adults (days 13 and 20, logFC range: −1.5 to −4.9, P < 0.05) compared to the reference group (Fig. 5B). One β-oxidation gene was upregulated on day 13 (logFC: 1.57, P = 0.03). However, a custom GO term manually assigned to all identified genes in β-oxidation pathway (GO:5000000, see methods) was not enriched among the upregulated (P = 0.815) or downregulated (P = 0.373) genes.

**Figure 6:**
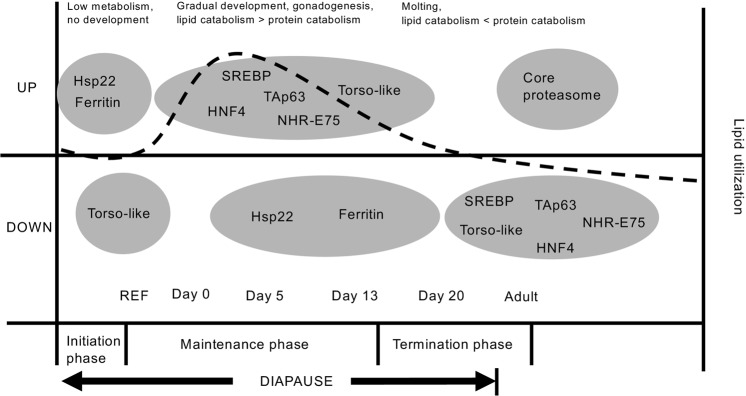
Hypothetical overall trend for expression of master regulators and lipid utilization during diapause, diapause termination and developmental progression in *Calanus finmarchicus.* The dashed line indicates the rate of lipid utilization. According to our observations, early *Calanus* spp. C5s have large stores of wax esters in their lipid sac during the intitation phase of diapause, and lipid catabolism (i.e. expression of β-oxidation genes) is low. Expression of ferritin, hsp22 and other genes related to increased stress tolerance is high, while expression of torso-like is low, and remains low in the beginning of the maintenance phase. Further into the maintenance phase, lipid catabolism increases. This coindices with high expression of torso-like and the master regulators of lipid metabolism SREBP, TAp63, HFN4 and NHR-E75. Closer to the termination of diapause, lipid catabolism declines and expression of these genes showed declining expression, reflecting lower energetic metabolism as the C5s are ready to molt into adults. When adulthood is reached under unfed conditions, both lipid catabolism and expression of the master regulator genes are low, and protein catabolism increases in order to maintain energy requirements.

Ferritin and hsp22, two proposed markers of *Calanus* diapause^30,31^, both exhibited significantly higher expression in the reference group compared to experimental C5s and adults at all time points (Fig. 6, and see Supplementary fig. S3 for details). Specifically, ferritin was downregulated compared to the reference group on day 0 (logFC: −1.85, F = 11.57, P < 0.01), day 5 (logFC: −2.06, F = 10.78, P < 0.01), day 13 (logFC: −2.73, F = 17.94, P < 0.01) and day 20 (logFC: −2.21, F = 7.15, P = 0.02). hsp22 was similarly downregulated from the reference group on day 0 (logFC: −3.06, F = 12.32, P < 0.01), day 5 (logFC: −4.51, F = 17.21, P < 0.01), day 13 (logFC: −4.90, F = 19.16, P < 0.01) and day 20 (logFC: −5.33, F = 11.31, P < 0.01). Within the experimental C5s, the expression of ferritin and hsp22 did not differ among any time points (P > 0.05). Torso-like, a proposed marker of developmental progression through the early portion of the C5 stage, showed increased expression in the experimental C5s on days 5 (logFC: 7.62, F = 4.90, P = 0.047), 13 (logFC: 7.61, F = 4.98, P = 0.048) and 20 (logFC: 8.92, F = 5.76, P = 0.034) compared to the reference group (Fig. 6 and Supplementary fig. S3). Expression of torso-like did not differ in C5s on day 0 compared to the reference group, nor among any of the time points in the experimental C5s (P > 0.05).

The “master regulators” of lipid metabolism SREBP, TAp63, HNF4 and NHR-E75 were generally expressed at higher levels in C5s than in adults (Fig. 6, also see Supplementary Table S5 for GLM metrics). For example, the GLM results suggest that NHR-E75 was higher in the reference group (logFC = 1.563, F = 15.03, *P* = 0.023), and in C5s on day 0 (logFC = 2.191, F = 28.12, *P* = 0.002), day 5 (logFC = 2.28, F = 27.46, *P* = 0.002), and day 13 (logFC = 1.819, F = 17.97, *P* = 0.013) compared to in adults on day 13. In order to assess whether the copepods needed to commence protein catabolism in addition to lipid catabolism^23^, we investigated gene expression of the core proteasome. Most of the core proteasome subunits were upregulated in adults compared to the reference group (Fig. 6, also see Supplementary Table S6). For example, proteosomal subunits β1-4 all exhibited elevated expressed levels in adults on day 13 (*P* > 0.05).

## Discussion

The primary objective of the present study was to investigate energetic metabolism in diapausing *Calanus* spp. C5s during progression to active adults. This was performed by manually annotating genes in the β-oxidation pathway within the *C. finmarchicus* transcriptome, and using RNA sequencing to investigate changes in expression of these genes during diapause termination and subsequent molting. Additionally, we investigated the expression of potential master regulators of lipid metabolism and molecular markers of diapause. General transcriptomic variation during the progression was also assessed.

The species determination analysis that was performed after sampling, pooling and gene expression analyses revealed the presence of *C. glacialis* together with *C. finmarchicus* in the Trondheimsfjord, at the depth of 400 m below the surface. The presence of both species at 400 m depth suggests that they may spend diapause together in this fjord. As will be discussed below, the patterns observed in the present study concerning lipid fullness ratio, energy metabolism and expression of target genes, do not seem to differ due to the species factor. Even though it has been recognized that the two species are genetically distinct^35^, *C. finmarchicus* and *C. glacialis* have similar life history traits, e.g. they both have wax esters as their primary storage lipids, and they have similar overwintering strategies^36^. It is therefore likely that similar gene expression patterns can be expected from these species. Thus, we can make cautious conclusions from these observations. Nevertheless, our results underline the importance of molecular species determinations for field-collected copepods and the need for future comparative studies.

All genes in the β-oxidation pathway in the KEGG database identified in *D. pulex* were successfully identified in *C. finmarchicus*, with the exception of CPT2 and BCD. In other organisms, the CPT2 enzyme functions along with CPT1 within the mitochondrial membrane^21^. The main function is to transport fatty acyl-CoA into the mitochondria for β-oxidation. Within each of three examined species of calanoid copepods (*C. finmarchicus, C glacialis* and *E. affinis*), we detected only a single CPT gene that was strongly supported as a CPT1 ortholog. The absence of a gene in a single transcriptomic database may reflect limitations in sequencing and assembly, but the absence of CPT2 from three species is more consistent with gene loss, some time after the divergence of the calanoids and harpacticoids. The calanoid CPT1 sequences were divergent and fell on long branches, which may reflect an expansion of CPT1 function in these species in association with the lack of CPT2; however, this hypothesis requires functional testing. Alternatively, other mitochondrial transporter enzymes may perform the functions usually performed by the carnitine shuttle (transport of fatty acids into mitochondria) in calanoid copepods. The presence of other possible alternative transporters should be subject for investigation in future studies of lipid catabolism in calanoid copepods.

We were unable to identify a BCD (EC 1.3.8.1) homolog in *C. finmarchicus*. This enzyme catalyzes the oxidation of a short-chain acyl-CoA to a short-chain trans-2,3-dehydroacyl-CoA using flavoprotein as electron acceptor^37^, and is involved in one of the later steps within the β-oxidation pathway in other species. Within the *C. finmarchicus* transcriptome, the transcript sequence most similar to BCD was annotated as a short-chain acyl-CoA dehydrogenase (EC 1.3.99.12). These two enzymes (BCD and EC 1.3.99.12) share an overlapping set of substrates and are involved in the same steps in β-oxidation, as well as other metabolic pathways. It is unknown what role, if any, the *C. finmarchicus* enzyme (EC 1.3.99.12) plays in β-oxidation, and further investigation is needed.

Mean lipid fullness ratio ranged from 22% in early diapause C5s (the reference group) to 12% in adults at the end of the experiment. As expected, the lipid fullness ratio was highest in the reference group as these C5s were in an early phase of diapause compared to the experimental copepods. The lipid fullness ratio was generally lower than otherwise reported in the literature for *Calanus* spp. C5s and adults^38–40^. The lower lipid fullness ratio may be explained by variation in individual feeding success^7,41^, as well as species-specific, geographical and seasonal variability in environmental conditions.

Mean lipid fullness ratio decreased over time during progression through the C5 stage (Fig. 2), indicating catabolism of lipids in the lipid sac to fuel metabolic functions. This is expected as most crustaceans preferentially catabolize lipids during periods of fasting or starvation^23^, and is similar to previous observations of diapausing *C. finmarchicus*^42^. There was no change over time in lipid fullness ratio in adults. This suggests that the adults may be using other sources to fuel energy requirements, or that adults have an overall lower requirement for energy. The adults may also have reduced their metabolism due to starvation as previously reported in copepods^43^.

Transcriptional profiles varied according to the developmental progression of the copepods with time, as shown by the first axis of the PCA (PC1, Fig. 3). In this PCA, the C5s in the reference group clustered to the far right, and adults to the far left. The intermediate and somewhat variable positioning of the experimental copepodites between the reference group and the adults along PC1 may suggest some asynchrony in development and diapause phase. It should again be noted that some of the C5 experimental samples contained *C. glacialis*. However, these samples were all positioned between the reference group and the adults, and did not appear to differ from the *C. finmarchicus* C5 experimental samples along PC1 and PC2. Furthermore, the samples containing *C. glacialis* do not diverge from the other samples with respect to expression of lipid catabolism genes or master regulators, as will be discussed below.

Developmental progression of the copepods was also reflected in the number of DEGs between the copepods on the different days (Fig. 4). The adults were the most different from the reference group, and the experimental C5s were intermediary between the reference group and adults. Together, these results illustrate the developmental progression occurring from C5 copepodites in early diapause to active adult copepods, i.e. the more time between the different sampling points, the more different the copepods are, with respect to number of DEGs.

When compared to the reference group, the most significantly enriched GO terms in the experimental C5s were dominated by terms related to development of gametes, cell division and molting. These were expected findings as these processes are important in preparations for adulthood and reproduction, and indicates that the experimental C5s were in various stages of development within the maintenance and termination phases of diapause. Similar processes were enriched in *N. flemingeri* in the weeks after emergence from diapause in a recent study^18^. The reference group had enriched GO terms mostly related to immune responses, suggesting a high resistance towards stressful stimuli, which is recognized as a diapause characteristic^7,18,30^. Put into the context of developmental progression, the reference group which was collected directly on the research vessel in August seems, as expected, to be in an earlier diapause stage, i.e. early maintenance phase, than the experimental C5s which were collected in November. Large variation in development between C5 copepodites has been previously observed in *C. finmarchicus* in a study by Tarrant *et al*.^17^. It is likely that some asynchrony in development and diapause termination must also occur in nature, to spread predation risk and to prolong phytoplankton feeding opportunities for offspring.

In this study, we treated the reference group as an initial developmental point (“time zero”) that could be compared with all the experimental copepods. Collection during two different seasons with potentially differing environmental conditions could be problematic. However, conditions such as temperature, salinity and pressure at 400 m depth remains very similar throughout the year at the sampling location (CTD-data, long term survey, Trondhjem Biological Station, database under contruction). Diapause itself can lead to a relatively homogenous metabolic state of the *Calanus* population at 400 m, with minimal access to food and predators for an extended time period. Thus, we argue that the results are likely not severely affected by sampling during different seasons, though it is desirable to repeat the experiment and make comparisons within a single season.

Although there was a linear decrease in lipid fullness ratio of C5s, the expression pattern of genes in the β-oxidation pathway (Fig. 5) suggests a non-linear utilization of lipids. Late C5s (days 13 and 20) had a higher number of downregulated β-oxidation genes compared to the reference group than early C5s (days 0 and 5). This suggests reduced lipid catabolism towards the end of diapause, i.e. close to diapause termination and molting (see Fig. 6 for hypothesized lipid utilization). During starvation, most crustaceans preferentially catabolize neutral lipids while conserving polar lipids, which have an essential role in maintaining cell membrane structure and integrity^23^. When the neutral lipid storage becomes depleted, proteins are increasingly utilized as an energy source^23,44^. Our results, which suggests a reduction in lipid catabolism towards diapause termination, could be explained by an adjustment during diapause from mainly lipid catabolism to a higher degree of protein degradation in late C5s. Similar distinct phases of biomass degradation has previously been reported in crusteceans^45^. Downregulation of β-oxidation genes was also observed in the adults (Fig. 5), and the adults sampled early in the experiment had similar mean lipid fullness ratio as those sampled late (Fig. 2). This suggests that the adjustment from primarily lipid catabolism toward increasing protein catabolism continued after the copepods reached adulthood. Indeed, several genes encoding the subunits of the proteasome were upregulated in the adults compared to the reference group, indicating increased protein catabolism. The lack of upregulation of these proteasome genes in the late C5s may indicate that low lipid stores could be an important endogenous cue for the initiation of moulting and development into adults, as has been previously suggested^46^.

Ferritin has been suggested to have a role in protection against oxidative stress and in delaying development in *C. finmarchicus*, and its expression has been hypothesized to be highest during the early stages of diapause^31^. Indeed, the expression of ferritin was highest in the reference group, and lower in C5s on days 0, 5, 13 and 20, and continued to decrease in adults (see Supplementary Fig. S3). As previously suggested, this implies that the reference group was in earlier stages of diapause than the experimental C5s, i.e. early maintenance phase. Similarly, hsp22 has been shown to be elevated in *C. finmarchicus* in diapause^30^, and suggested to be involved in stress tolerance. In the present study, this gene had a similar expression pattern as ferritin, and showed a trend of reduction during termination of diapause. Torso-like protein has been suggested as a good discriminator between early and late C5s regarding development, with increasing expression with progression throughout the C5 stage^17^. This protein helps to regulate both developmental timing and body size^17,47^. Torso-like expression was low in the reference group and in C5s on day 0, and higher in later C5s, before dropping in the adults. This implies that after day 0 the experimental C5s were further along in development.

Expression of the master regulators was generally upregulated in early C5s (the reference group, and days 0 and 5) and downregulated in adults and/or late C5s (days 13 and 20). SREBP is a membrane-bound transcription factor that can stimulate lipid biosynthesis, as shown in insects, where it has an essential role in membrane production^26^. TAp63 is closely linked to lipid metabolism in mice^25^. HNF4 belongs to a subfamily of nuclear receptors (NR2A), which is represented by a single gene in *Drosophila* and has further duplicated within vertebrate and nematode lineages. In *Drosophila*, HNF4 regulates genes needed for β-oxidation and lipid mobilization^28^. NHR-E75 is a member of the same nuclear receptor family as the ecdysone receptor (EcR; NR1D and NR1H, respectively), E75 has been identified in many insect species^48^. E75 is involved in molting and developmental progression in arthropods^27,48^, and could therefore be expected to be upregulated in the late C5 which were close to the terminal molt. It has also been suggested that in *D. melanogaster* E75 may be a functional equivalent of the vertebrate PPARγ, which is a master regulator of lipid metabolism^49^. It is therefore possible that E75 could be linked to the lipid metabolism in copepods, and that the downregulation in the late C5s is related to the downregulation of β-oxidation genes in these copepods observed in the present study.

Together, the expression profiles of the molecular markers of diapause/development and the master regulators suggest that the reference group was comparatively early in the maintenance phase, while the other early C5s (i.e. days 0 and 5) were in the second half of the maintenance phase, where development is gradually initiated^15^ and fueled by catabolism of wax esters in the lipid sac. Late C5s were closer to adulthood, which was reflected by the expression of master regulators of lipid metabolism that did not differ compared to the adults (see Supplementary Table S5). Hypothesized trends based on the observed gene expression and lipid catabolism profiles are illustrated in Fig. 6. Following our observations, early *Calanus* spp. C5s have large lipid sacs during the initiation phase of diapause, as well as very low lipid catabolism and high expression of ferritin, hsp22 and other genes related to increased enzyme and nutrient conservation and increased stress tolerance. Torso-like expression is low in early diapause (the initiation phase) and in the beginning of the maintenance phase (reference group and C5s from day 0 in the present study). Later in the maintenance phase, the C5s catabolize wax esters from their lipid sacs. This catabolism declines with developmental progression during diapause. High lipid catabolism is expected to occur during energetically-demanding stages of development, one of which, according to our observations, seems to transpire relatively early during the maintenance phase (early C5s), and not during preparation for molting during the termination phase. Elevated expression of torso-like, SREBP, TAp63, HFN4 and NHR-E75 was observed in the early C5s, and can thus be interpreted to coincide with increased energetic metabolism and intiation of developmental processes. Closer to the termination phase (late C5s), these genes showed declining expression, reflecting lower energetic metabolism and lipid catabolism as the C5s are ready to molt into adults. When adulthood is reached under unfed conditions, both lipid catabolism and expression of the master regulator genes are low, and protein catabolism is increased in order to maintain energy requirements.

Future research regarding energetic metabolism during diapause in *C. finmarchicus* should include attempts to induce diapause in the laboratory, to circumvent the unavoidable disturbance from collection of copepods in diapause and to avoid the presence of other *Calanus* species. Despite the apparent similarities in genetic variation during diapause termination of *C. finmarchicus* and *C. glacialis*, targeted comparisons of diapause physiology between the two species should be performed. The improved annotation of the genes in the β-oxidation pathway provides a foundation for further exploration of basic physiology in these keystone species in northern marine ecosystems. Our findings also provide a basis for a better understanding of the development occurring during diapause and diapause termination, and can in this way contribute to understand how large-scale changes, such as climate change, can influence copepod population dynamics and whole marine ecosystems.

## Methods

### Copepod collection

*Calanus* spp. copepodites of stage C5 were collected from sea bed depth (400 m) up to 200 m in the Trondheimsfjord, Norway (N63°29′, E10°18) using a Nansen net (mesh size 200 μm) with a closing mechanism and a non-filtering cod end^50^. In this fjord, *C. finmarchicus* C5s migrate to deep waters to diapause in May, and resurface to reproduce in March^51^. Collection of copepodites in early stages of diapause was performed directly on the research vessel in August 2017, and will henceforth be referred to as “the reference group”. Collection of C5s (“the experimental C5s”) later in the diapause phase was performed in November 2016 from the same vessel. During sampling of both groups, the net was hauled at a speed of approximately 0.3 m/s to reduce physical stress to the copepods. On deck, the cod end was quickly removed from the net after retrival, and the content was screened on a 1300 μm mesh sieve submerged in a bucket of seawater. In this process, chaetognaths, significant copepod predators^52^, were eliminated from the samples. The copepods were temporarily kept in filtered seawater (salinity: ~33‰, temperature range 4–9 °C) in 30 L buckets that were covered in three layers of dark plastic, until they were either sampled for RNA seq (the reference group) on the research vessel, or transported (the experimental group) to the laboratory facilities on shore (NTNU Sealab, Trondheim, Norway). During sampling, the copepods were euthanized with tricaine methane-sulfonate (Finquel, 1.5 g/L seawater, Argent Laboratories Redmond, WA, USA) and photographed using CCD still-video camera (Sony DWF-sx900, Sony Corporation, Tokyo, Japan) operated by Fire-i software (Unibrain, Inc., San Ramon, CA, USA) connected to a dissecting microscope (Leica Microsystems, Germany). The frames were stored on a PC for further processing. For sampling of the reference group, 10 individuals per sample (n = 2) were placed together in 1 mL RNA later (ThermoFisher, USA) in 1.5 mL microcentrifuge tubes, which were refrigerated at 4 °C for approximately 24 hours before they were moved to −20 °C.

After transportation to the laboratory facilities on shore, the experimental C5s were acclimated in two 250 L polystyrene tanks with constant flow-through of filtered natural seawater (170 mL/min, 8 °C, no light exposure) for 13 days before the experiment was initiated. This was to ensure acclimatization to laboratory conditions, and to minimize continuous stimulation prior to the onset of experiment. The copepods were not fed for the duration of the experiment.

### Experimental setup

To initiate the experiment, the copepods were temporarily sorted from the 250 L tanks using ladles and a 64 μm sieve into plastic cups, and then into the experimental 5 L borosilicate jars (n = 4) with open tops and modified spouts, which ensures an even outflow of water matching the continuous inflow of ~12 mL/min of filtered natural seawater. All handling was performed at 8 °C, using a halogen flashlight with a red glass filter for illumination. The room was kept in the dark and at 8 °C, and the experimental containers were kept behind light-blocking curtains throughout the experiment.

Each sampling day, three C5s per replicate were sampled for RNA seq in the same way as described above for the reference group. The first sampling of C5s in the experiment was performed on the same day as the sorting (day 0, 13 days after collection, Table 1). The second was performed on day 5, the third and fourth sampling were performed on days 13 and 20. On days 13 and 20, adults appeared in sufficient number for sampling, so three adults per replicate (females only) were sampled in addition on these days.

### Species determination

Recently, the occurrence of the “true Arctic shelf species” *C. glacialis*^3^ was documented in the Trondheimsfjord through molecular analyses^32,33^. This information became available after the sampling and sequencing for the present study had already been performed. Morphological criteria have recently been deemed insufficient to reliably distinguish between *C. glacialis* and *C. finmarchicus*^32^. Thus, molecular-based species identification was performed to identify the potential presence of *Calanus* species other than *C. finmarchicus* in aliquots of the RNA samples, which consisted of RNA from pooled individuals. Archived RNA aliquots from all samples sequenced were used to investigate the potential presence of *C. glacialis* among the samples. First, RNA extracts of pooled individuals were converted into first-stranded cDNA using the Invitrogen™ SuperScript ™ II Reverse Transcriptase (Life Technologies) following the manufacturer’s protocol. This cDNA was then used as template for molecular species identification following the procedure previously described^33^, based on 5 InDel-type markers^53^ (only the transcriptome-based markers were used).

### Biometry

The length, height and area of each copepod’s prosome and lipid sac were estimated from the photographs using ImageJ software (version 1.48k) with a drawing tablet (Wacom, Co. Ltd. Japan). Developmental stage (C5 and adult) was also determined from the photographs. Lipid sac volume and prosome volume were estimated using methods similar to Miller *et al*.^41^. Lipid fullness ratio (%) was calculated as the ratio of (lipid sac volume/prosome volume) * 100.

Statistical analyses of biometrical data were performed in R (Version 3.3.1). Outliers were removed using Tukey’s method where data points above or below the 1.5 x interquantile range are removed. Two outliers were removed, leaving n = 319. The effects of time (as days since start of experiment) and stage (C5, n = 185 and adults n = 136) on lipid fullness ratio were investigated using an ANCOVA with time and stage as explanatory variables. Visual inspection of the model residuals confirmed their normal distribution. The reference group was excluded from the ANCOVA as this group was sampled directly after collection in the field, and thus not subjected to acclimation like the experimental copepods were, leading to a much higher range of lipid fullness ratios.

### RNA isolation, library preparation and RNA sequencing

Samples were selected for RNA sequencing based on the median lipid fullness ratio of all samples were chosen for RNA sequencing. This selection consisted of two or three replicates from each day that C5s were sampled (reference and days 0, 5, 13 and 20), and two replicates of adults from days 13 and 20. Prior to RNA extraction, the copepods from each sample were removed from RNAlater, gently dried on a paper towel and weighed. Total RNA extraction was performed using the Qiagen RNeasy Plus Universal Mini Kit (Qiagen Inc., Valencia, CA, USA) with additional use of a QiaShredder column, following the manufacturer’s protocol. The RNeasy kit includes a genomic DNA removal step. Final eluation volume of RNA was 30 μL. RNA quality was assessed using a Model 2100 Bioanalyzer instrument (Agilent, Santa Clara, USA). Meaningful RNA Integrity (RIN) values could not be obtained because the hydrogen bonds in arthropod 28S rRNA are disrupted in the denaturation heating step prior to Bioanalyzer analysis, resulting in fragments that migrate closely with 18S rRNA^54^. RNA quality was therefore assessed using electrophoregram summary graphs and gel images. All analyzed samples were of high quality, containing a strong 18S band and little or no evidence of genomic DNA contamination (large bands) or degradation (smear of smaller bands).

For RNA sequencing, cDNA libraries were synthesized from total RNA (40 ng/μL input) using the Illumina TruSeq Stranded mRNA sample preparation kit (Illumina, San Diego, USA), with minor modifications adjusted for smaller volumes than in the manufacturer´s protocol. Final volumes of cDNA libraries were 22 μL. Prior to RNA sequencing, the cDNA libraries were pooled and normalized, and a quality control was performed on a Bioanalyzer instrument by the sequencing facility. Pooling was conducted in order to incorporate biological material from a larger number of individuals into a limited number of RNA-seq libraries^55^, as is common in gene expression studies with copepods (reviewed by Tarrant *et al*.^56^).

### Illumina sequencing and bioinformatic analyses

Samples were sequenced at the Genomic Core Facility (GCF) at NTNU, Trondheim, with 75 bp paired-end reads on an Illumina HiSeq 500 HO flowcell. This produced ~130 million reads in total, and ~9 million reads per sample (see Supplementary Table S7). Each sample was sequenced on four lanes on the Illumina flow cell. Adapter sequences were trimmed at the GCF. Quality control was performed with MultiQC (version 1.2.dev0).

The present study focuses primarily on *C. finmarchicus*, as this species has been considered to be the dominant zooplankton species in the Norwegian Sea^57^. Thus, a *C. finmarchicus* reference transcriptome (PRNJA231164) was used as the main genomic database for the bioinformatic analyses. The reference transcriptome was re-annotated in its entirety^58^ (10.6084/m9.figshare.8199416.v1) using Trinotate (version 3.0)^59^. Within Trinotate, transcripts were compared against Swiss-Prot using blastx (version 2.2.30). This information was then used by Trinotate to assign GO terms and KEGG orthology (KO) groups.

Read mapping and transcript abundance estimation were performed using scripts bundled within Trinity (version 2.0.6)^60^, as previously described in Skottene *et al*. (2019^61^), i.e. Samtools (version 0.1.19) and Bowtie (version 1.0.0). Reads were mapped to the reference transcriptome and quantified using the RSEM package (version 1.2.12)^62^. Read counts were normalized to trimmed mean of M-values (TMM) to account for difference in library sizes, and FPKM-normalized (fragments per feature kilobase per million reads mapped) when producing PCA plots, using scripts within the Trinity pipeline. Differentially expressed genes (DEGs) between stages (adult vs C5) on days 0, 5, 13 and 20 compared to the reference group were analysed with generalized linear models (GLM) with a negative binomial distribution using packages EdgeR (version 3.6.8) and limma (version 3.20.9) within Bioconductor in R Version 3.2.3^63^. Alpha level was set to 0.05. Expected counts from RSEM were used as input in the GLM. Genes with very low counts per million (CPM ≤ 1) were filtered out. Tagwise dispersion was calculated using the Cox-Reid profile-adjusted likelihood method, which allows for multiple factors in the GLM^64^. EdgeR uses the Benjamini-Hochberg method for adjusting p-values for multiple comparisons. In a separate analysis, the reference group was compared to all C5s of the experiment combined, using the exact test in EdgeR, where dispersion is calculated using the quantile-adjusted conditional maximum likelihood method. This analysis was the basis for the gene ontology (GO) enrichment analysis, which was performed on up- and downregulated DEGs separately, using the goseq (version 1.26.0)^65^ and GO.db (version 3.4.0)^66^ packages in R (version 3.3.1) (Rstudio version 1.1.383).

To further improve the annotation of the *C. finmarchicus* transcriptome, we manually annotated genes in the β-oxidation pathway, using as a reference the amino acid sequences of enzymes in the β-oxidation pathway from *D. pulex*, the only crustacean in the KEGG database as of December 2017 (dpx00071). It was also the species with most top hit blastx searches for *C. finmarchicus* in the study by Lenz *et al*.^16^. A nucleotide database was made from the reference transcriptome, and each amino acid sequence was used in a tblastx (BLAST+, version 2.7.1) query (translated nucleotide sequence toward amino acid sequence). E-value cutoff was set at 10^−7^. Following the manual annotation of the genes in the β-oxidation pathway, we observed that the automatic blast-based annotation had not consistently assigned these genes to a common GO term or pathway (i.e., related to β-oxidation). Similar limitations of automated blast-based annotation of the *C. finmarchicus* transcriptome have previously been observed for the glycolysis pathway^67^. To assess GO enrichment of the β-oxidation pathway specifically, we manually assigned all the genes with a custom term, “GO:5000000,” and repeated the enrichment analysis on up- and downregulated genes separately, comparing the experimental C5s combined against the reference group.

The same method as described above was used (with tblastn) when identifying master regulators of lipid metabolism and protein degradation genes (proteasome). The amino acid sequence for SREBP was obtained from *Daphnia magna* (SI, Table S8). Two partial sequences encoding TAp63 were obtained from *Mytilus galloprovincialis*, and both sequences had the same top hit in *C. finmarchicus*. The amino acid sequence for HNF4 was obtained from *T. japonicus*. Due to the importance of the peroxisome proliferator activator receptor (PPAR) as a master regulator of energy and lipid homeostasis in many other species^68^, we investigated the presence of this receptor in *C. finmarchicus*. The top hit had a 35% identity with a sequence that was annotated as NHR-E75. Amino acid sequences encoding all subunits of the core proteasome were obtained from *D. melanogaster*.

A phylogenetic analysis was conducted to investigate the apparent loss of a CPT gene from *C. finmarchicus*. CPT1 and CPT2 amino acid sequences were retrieved from the KEGG database (EC: 2.3.1.21) for *D. pulex, D. melanogaster, T. castaneum, H. sapiens*, and *M. musculus* (accession numbers for amino acid sequences in Supplementary Table S9a). We used the *D. pulex* sequences to search transcriptomic databases for the copepod species *C. glacialis* (PRJNA237014, PRJNA274584)*, E. affinis* (PRJNA242763), and *T. japonicas* (PRJNA274317) using tblastn with default parameters (nucleotide sequence accession numbers in Supplementary Table S9b). We also searched a second transcriptomic database that had been independently assembled for *C. finmarchicus* (PRJNA236528). The longest open reading frame was identified within each copepod transcript using a biopython-based script^69,70^ (reported in Supplementary Table S10). All sequences were aligned using MUSCLE with default parameters^71^. A maximum likelihood analysis was conducted using RAXML-HPC version 8 with rapid bootstrapping options^72^, as implemented on the XSEDE platform through the CIPRES Science Gateway^73^. Trees were visualized and edited (font size, position of apparent root) using FigTree version 1.1.2 and Adobe Illustrator (version CS4, repositioning of labels for visibility, labelling of clades).

## Supplementary information

Supplementary Information: The β-oxidation pathway is downregulated during diapause termination in Calanus copepods

## Data Availability

Sequence data have been submitted to the National Center of Biotechnology Information (NCBI; www.ncbi.nlm.nih.gov) under the Bioproject PRJNA420690.
