# Supplementary material for: The β-oxidation pathway is downregulated during diapause termination in Calanus copepods
Source: Sci Rep. 2019 Nov 13;9:16686. doi: 10.1038/s41598-019-53032-5 (PMC6853931; doi:10.1038/s41598-019-53032-5)
Supplement: Supplementary file 1 — Supplementary Information: The β-oxidation pathway is downregulated during diapause termination in Calanus copepods [file 41598_2019_53032_MOESM1_ESM.docx]

**Supplementary Information: The β-oxidation pathway is downregulated during diapause termination in *Calanus* copepods**

Elise Skottene^1*^, Ann M. Tarrant^2^, Anders J. Olsen^1^, Dag Altin^3^, Mari-Ann Østensen^1^, Bjørn Henrik Hansen^4^, Marvin Choquet^5^, Bjørn M. Jenssen^1^ and Rolf Erik Olsen^1^

^1^Department of Biology, NTNU, Trondheim, Norway; ^2^Woods Hole Oceanographic Institution, Woods Hole, Massachusetts, USA; ^3^BioTrix, Trondheim, Norway; ^4^SINTEF Ocean AS, Environment and New Resources, Trondheim, Norway; ^5^Faculty of Biosciences and Aquaculture, Nord University, Bodø, Norway

*Corresponding author, email: elise.skottene@ntnu.no

**Content**

Table S1. Distribution of the two species *Calanus finmarchicus* and *C. glacialis* in all RNA-seq

samples. Sample determination analyses using InDel markers was performed on RNA aliquots

containing pooled RNA from three (experimental group) or ten (reference group) individuals. Ad: adults. ref: reference group.

Table S2. Identification of genes in the β-oxidation pathway in the *Calanus finmarchicus* transcriptome based on amino acid sequences from *Daphnia pulex.*

Table S3. Number of differentially expressed genes (DEGs, P<0.05) between comparisons of *Calanus* spp. C5s and adults sampled at all time points within the experiment. C5: C5 copepodites. AD: adults.

Table S4. Differential expression (log2 fold change (FC), log2 counts per million (CPM), F value, P-value and false discovery rate (FDR) of genes in the β-oxidation pathway of *Calanus* spp. C5s sampled at day 0, 5, 13 and 20 and adults from day 13 and 20 compared to the reference group (early diapause C5s). Analysed with generalized linear models (GLM) in EgdeR.

Table S5. Differential expression of master regulator genes identified in *Calanus* C5s and adults sampled at all time points within the experiment.

Table S6. Top hit sequences and differential expression of proteasome subunits identified in *Calanus* C5s and adults sampled at all time points within the experiment.

Sequences were obtained from *Drosophila melanogaster.*

Table S7. Number of reads per sample. NCBI accession numbers assigned to each sample library. Ad: adults. ref: reference group. M seqs: number of reads per sample library in millions.

Table S8a. NCBI accession numbers for amino acid sequences encoding carnitine palmitoyltransferase 1 (CPT1) and 2 (CPT2), retrieved from the KEGG database.

Table S8b. NCBI accession number for nucleotide sequences encoding carnitine palmitoyltransferase 1 (CPT1) and 2 (CPT2), based on *Daphnia pulex* sequences, from transcriptomic databases of copepod species.

Table S9. Predicted open reading frames for copepod CPT sequences.

Figure S1. Most statistically significantly enriched GO terms (lowest P-values) in the reference group compared to copepodites from day 0, 5, 13 and 20 combined. MF: Molecular function. CC: Cell component. BP: Biological process.

Figure S2. Most statistically significantly enriched GO terms (lowest P-values) in the copepodites from day 0, 5, 13 and 20 combined compared to the reference group. MF: Molecular function. CC: Cell component. BP: Biological process.

Figure S3. Differential expression (log2 fold change) of molecular markers of diapause and development in *C. finmarchicus* copepodites sampled at day0 (T0), 5 (T1), 13 (T2) and 20 (T3) and adults from day 13 and 20, compared to the reference group. A log2 fold change of -2 signifies a 4 fold decrease from the reference group

**Table S1.** Distribution of the two species *Calanus finmarchicus* and *C. glacialis* in all RNA-seq samples. Sample determination analyses using InDel markers was performed on RNA aliquots containing pooled RNA from three (experimental group) or ten (reference group) individuals. Ad: adults. ref: reference group.

| **Stage** | **Day** | **Replicate** | ***C. finmarchicus*** | ***C. glacialis*** | **mix** |
| --- | --- | --- | --- | --- | --- |
| C5 | ref | 1 |  |  | X |
| C5 | ref | 2 |  |  | X |
| C5 | 0 | 1 | X |  |  |
| C5 | 0 | 2 |  |  | X |
| C5 | 0 | 3 |  |  | X |
| C5 | 5 | 1 | X |  |  |
| C5 | 5 | 2 | X |  |  |
| C5 | 13 | 1 | X |  |  |
| C5 | 13 | 2 | X |  |  |
| C5 | 20 | 1 |  | X |  |
| AD | 13 | 1 | X |  |  |
| AD | 13 | 2 |  |  | X |
| AD | 20 | 1 | X |  |  |
| AD | 20 | 2 |  |  | X |

**Table S2**. Identification of genes in the β-oxidation pathway in the *Calanus finmarchicus* transcriptome based on amino acid sequences from *Daphnia pulex.*

| **EC number** |  |  | **Top hit in *D. pulex*** | |  |
| --- | --- | --- | --- | --- | --- |
| **Enzyme name in KEGG** | **GeneID** | **Annotation** | **% identity** | **E-value** | **Accession** |
| EC 6.2.1.3: Long-chain-fatty-acid-CoA ligase | comp273007_c0_seq2 | Long-chain-fatty-acid--CoA ligase 1 | 52.1 | 0.00E+00 | Q7ZYC4.1 |
|  | comp271267_c0_seq2 | Long-chain-fatty-acid--CoA ligase 4 | 49 | 0.00E+00 | O35547.1 |
|  | comp274130_c0_seq1 | Long-chain-fatty-acid--CoA ligase 1 | 55.9 | 0.00E+00 | Q9JID6.1 |
| EC 2.3.1.21: Carnitine O-acetyltransferase 1 | *comp272598_c0_seq5* | Carnitine O-acetyltransferase | 31.3 | 8.21E-64 | P52826.1 |
|  |  |  |  |  |  |
| EC 2.3.1.21: Carnitine O-acetyltransferase 2 | *comp272598_c0_seq5* | Carnitine O-acetyltransferase | 27.3 | 2.59E-65 | P52826.1 |
|  |  |  |  |  |  |
|  |  |  |  |  |  |
| EC 1.3.3.6: acyl-CoA oxidase | comp270252_c0_seq2 | Peroxisomal acyl-coenzyme A oxidase 3 | 45.3 | 0.00E+00 | O15254.2 |
|  | comp256748_c0_seq1 | Probable peroxisomal acyl-coenzyme A oxidase 1 | 48.8 | 0.00E+00 | Q7KML2.1 |
|  |  |  |  |  |  |
| EC 1.3.8.7: acyl-CoA dehydrogenase | comp267356_c1_seq1 | Probable medium-chain specific acyl-CoA dehydrogenase, mitochondrial | 75.4 | 0.00E+00 | Q9VSA3.1 |
|  |  |  |  |  |  |
| EC 1.3.8.9: very long chain acyl-CoA dehydrogenase | comp269157_c0_seq1 | Very long-chain specific acyl-CoA dehydrogenase, mitochondrial | 61.8 | 0.00E+00 | P45953.1 |
|  |  |  |  |  |  |
| EC 1.3.8.8: long-chain-acyl-CoA dehydrogenase | comp264820_c0_seq1 | Long-chain specific acyl-CoA dehydrogenase, mitochondrial | 57.6 | 1.02E-168 | P79274.1 |
|  |  |  |  |  |  |
| EC 4.2.1.17: enoyl-CoA hydratase | comp260899_c0_seq1 | Enoyl-CoA hydratase, mitochondrial | 64.3 | 1.71E-112 | Q8BH95.1 |
|  |  |  |  |  |  |
| EC 4.2.1.17: 1.1.1.211: enoyl-CoA hydratase / long-chain 3-hydroxyacyl-CoA dehydrogenase | comp275258_c0_seq2 | Trifunctional enzyme subunit alpha, mitochondrial | 60.2 | 0.00E+00 | P40939.2 |
|  |  |  |  |  |  |
|  |  |  |  |  |  |
| EC 1.1.1.35: 3-hydroxyacyl-CoA dehydrogenase | comp261049_c0_seq1 | Hydroxyacyl-coenzyme A dehydrogenase, mitochondrial | 62.6 | 8.51E-132 | Q9WVK7.1 |
|  |  |  |  |  |  |
| EC 2.3.1.16: acetyl-CoA acyltransferase | comp271572_c0_seq1 | Trifunctional enzyme subunit beta, mitochondrial | 73.6 | 0.00E+00 | Q99JY0.1 |
|  |  |  |  |  |  |
| EC 2.3.1.16: acetyl-CoA acyltransferase 2 | comp262743_c0_seq1 | 3-ketoacyl-CoA thiolase, mitochondrial | 64.9 | 0.00E+00 | P42765.2 |
|  |  |  |  |  |  |
| EC 1.3.8.1: butyryl-CoA dehydrogenase | *comp266079_c0_seq1* | Short-chain specific acyl-CoA dehydrogenase, mitochondria | 38.1 | 3.50E-84 | P15651.2 |
|  |  |  |  |  |  |
| EC 1.3.99.12: short/branched chain acyl-CoA dehydrogenase | *comp266079_c0_seq1* | Short-chain specific acyl-CoA dehydrogenase, mitochondrial | 38.1 | 3.50E-84 | P15651.2 |
|  |  |  |  |  |  |
| EC 1.3.8.6: glutaryl-CoA dehydrogenase | comp271450_c0_seq2 | Glutaryl-CoA dehydrogenase, mitochondrial | 72.7 | 0.00E+00 | Q2KHZ9.1 |
|  |  |  |  |  |  |
| EC 2.3.1.9: acetyl-CoA C-acetyltransferase | comp266860_c1_seq2 | Acetyl-CoA acetyltransferase, mitochondrial | 64.5 | 0.00E+00 | Q5BKN8.1 |
|  |  |  |  |  |  |
| EC 5.3.3.8: 3,2-trans-enoyl-CoA isomerase, mitochondrial | comp271999_c0_seq2 | Enoyl-CoA delta isomerase 1, mitochondrial | 51.6 | 1.10E-82 | P42125.2 |
|  | comp268447_c0_seq2 | Enoyl-CoA delta isomerase 2, mitochondrial | 50 | 9.97E-85 | O75521.4 |
|  |  |  |  |  |  |
| EC 1.1.1.284: 1.1.1.1: S-(hydroxymethyl)glutathione dehydrogenase / alcohol dehydrogenase | comp273523_c0_seq4 | Alcohol dehydrogenase class-3 | 59.8 | 8.14E-134 | Q17335.1 |
|  | comp257585_c0_seq2 | Alcohol dehydrogenase class-3 | 44.5 | 7.16E-88 | Q17335.1 |
|  |  |  |  |  |  |
|  |  |  |  |  |  |
| EC 1.2.1.3: aldehyde dehydrogenase (NAD+) | comp269979_c0_seq2 | Aldehyde dehydrogenase X, mitochondrial | 67.4 | 0.00E+00 | Q66HF8.1 |
|  | comp262846_c0_seq1 | Aldehyde dehydrogenase, mitochondrial | 71.4 | 0.00E+00 | P11884.1 |
|  | comp268106_c0_seq1 | Aldehyde dehydrogenase family 3 member B1 | 49.1 | 3.74E-142 | Q5XI42.1 |
|  | comp269490_c0_seq2 | Alpha-aminoadipic semialdehyde dehydrogenase | 71.3 | 8.26E-139 | Q2KJC9.4 |
| EC 1.2.1.47: 1.2.1.3: aldehyde dehydrogenase family 9 member A1 | comp270229_c0_seq1 | Aldehyde dehydrogenase family 9 member A1-A | 54.3 | 0.00E+00 | Q7ZVB2.1 |
|  |  |  |  |  |  |
|  |  |  |  |  |  |

**Table S3.** Number of differentially expressed genes (DEGs, P<0.05) between comparisons of *Calanus* spp. C5s and adults sampled at all time points within the experiment. C5: C5 copepodites. AD: adults.

| **Comparison** | **Upregulated** | **Downregulated** | **Total** |
| --- | --- | --- | --- |
| C5_Day0vsRef | 2496 | 2596 | 5092 |
| C5_Day5vsRef | 2301 | 1979 | 4280 |
| C5_Day13vsRef | 2876 | 2551 | 5427 |
| C5_Day20vsRef | 1737 | 2031 | 3768 |
| C5_Day5vsDay0 | 619 | 499 | 1118 |
| C5_Day13vsDay0 | 684 | 680 | 1364 |
| C5_Day20vsDay0 | 728 | 1198 | 1926 |
| C5_Day13vsDay5 | 416 | 495 | 911 |
| C5_Day20vsDay5 | 885 | 1146 | 2031 |
| C5_Day20vsDay13 | 849 | 1006 | 1855 |
| Ad_Day13vsRef | 5000 | 5580 | 10580 |
| Ad_Day20vsRef | 4255 | 5473 | 9728 |
| Ad_Day13vsC5_Day0 | 3000 | 3896 | 6896 |
| Ad_Day20vsC5_Day0 | 2392 | 4059 | 6451 |
| Ad_Day13vsC5_Day5 | 2689 | 4225 | 6914 |
| Ad_Day20vsC5_Day5 | 2118 | 4223 | 6341 |
| Ad_Day20vsC5_Day13 | 719 | 2349 | 3068 |
| Ad_Day13vsC5_Day13 | 1087 | 2487 | 3574 |
| Ad_Day20vsC5_Day20 | 1840 | 2423 | 4263 |

**Table S4.** Differential expression (log2 fold change (FC), log2 counts per million (CPM), F value, P-value and false discovery rate (FDR) of genes in the β-oxidation pathway of *Calanus* spp. C5s sampled at day 0, 5, 13 and 20 and adults from day 13 and 20 compared to the reference group (early diapause C5s). Analysed with generalized linear models (GLM) in EgdeR.

| **Stage** | **Day** | **Gene ID** | **EC** | **Enzyme name Trinotate** | **logFC** | **logCPM** | **F** | **P** | **FDR** |
| --- | --- | --- | --- | --- | --- | --- | --- | --- | --- |
| C5 | 0 | comp261049_c0 | EC:1.1.1.35 | Hydroxyacyl-coenzyme A dehydrogenase, mitochondrial | -1.403 | 7.004 | 10.818 | 0.027 | 0.247 |
|  | 0 | comp262846_c0 | EC:1.2.1.3 | Aldehyde dehydrogenase, mitochondrial | -1.934 | 7.852 | 25.003 | 0.002 | 0.079 |
|  | 0 | comp266860_c1 | EC:2.3.1.9 | Acetyl-CoA acetyltransferase, mitochondrial | -1.294 | 6.019 | 7.669 | 0.045 | 0.305 |
|  | 0 | comp271341_c0 | EC:6.2.1.3 | Long-chain-fatty-acid--CoA ligase ACSBG2 | 4.289 | 5.410 | 15.585 | 0.002 | 0.070 |
| C5 | 5 | comp262350_c0 | EC:1.1.1.284 1.1.1.1 | Alcohol dehydrogenase class-3 chain L | -2.713 | 2.101 | 5.477 | 0.038 | 0.350 |
|  | 5 | comp262846_c0 | EC:1.2.1.3 | Aldehyde dehydrogenase, mitochondrial | -1.705 | 7.852 | 15.092 | 0.018 | 0.293 |
|  | 5 | comp271341_c0 | EC:6.2.1.3 | Long-chain-fatty-acid--CoA ligase ACSBG2 | 4.388 | 5.410 | 15.309 | 0.002 | 0.128 |
| C5 | 13 | comp257585_c0 | EC:1.1.1.284 1.1.1.1 | S-(hydroxymethyl)glutathione dehydrogenase | -1.817 | 5.435 | 15.719 | 0.017 | 0.201 |
|  | 13 | comp261049_c0 | EC:1.1.1.35 | Hydroxyacyl-coenzyme A dehydrogenase, mitochondrial | -1.862 | 7.004 | 14.233 | 0.011 | 0.168 |
|  | 13 | comp262350_c0 | EC:1.1.1.284 1.1.1.1 | Alcohol dehydrogenase class-3 chain L | -2.873 | 2.101 | 5.178 | 0.043 | 0.282 |
|  | 13 | comp262846_c0 | EC:1.2.1.3 | Aldehyde dehydrogenase, mitochondrial | -1.626 | 7.852 | 13.673 | 0.025 | 0.235 |
|  | 13 | comp263616_c0 | EC:4.2.1.17 | Probable enoyl-CoA hydratase, mitochondrial | -4.610 | 0.634 | 8.783 | 0.035 | 0.264 |
|  | 13 | comp271341_c0 | EC:6.2.1.3 | Long-chain-fatty-acid--CoA ligase ACSBG2 | 4.040 | 5.410 | 13.259 | 0.004 | 0.096 |
|  | 13 | comp271450_c0 | EC:1.3.8.6 | Glutaryl-CoA dehydrogenase, mitochondrial | -1.502 | 6.150 | 10.624 | 0.042 | 0.282 |
|  | 13 | comp274130_c0 | EC:6.2.1.3 | Long-chain-fatty-acid--CoA ligase 1 | -1.952 | 3.694 | 6.379 | 0.029 | 0.248 |
| C5 | 20 | comp261049_c0 | EC:1.1.1.35 | Hydroxyacyl-coenzyme A dehydrogenase, mitochondrial | -2.024 | 7.004 | 9.719 | 0.036 | 0.396 |
|  | 20 | comp262743_c0 | EC:2.3.1.16 | 3-ketoacyl-CoA thiolase, mitochondrial | -2.708 | 7.713 | 27.500 | 0.008 | 0.274 |
|  | 20 | comp262846_c0 | EC:1.2.1.3 | Aldehyde dehydrogenase, mitochondrial | -2.282 | 7.852 | 14.847 | 0.019 | 0.334 |
|  | 20 | comp263640_c0 | EC:4.2.1.17 | Methylglutaconyl-CoA hydratase, mitochondrial | -1.984 | 6.901 | 18.645 | 0.042 | 0.408 |
|  | 20 | comp269490_c0 | EC:1.2.1.3 | Alpha-aminoadipic semialdehyde dehydrogenase | -7.699 | 3.289 | 7.924 | 0.016 | 0.318 |
|  | 20 | comp271450_c0 | EC:1.3.8.6 | Glutaryl-CoA dehydrogenase, mitochondrial | -3.097 | 6.150 | 21.556 | 0.004 | 0.221 |
|  | 20 | comp273007_c0 | EC:6.2.1.3 | Long-chain-fatty-acid--CoA ligase ACSBG2 | -2.199 | 5.678 | 12.245 | 0.032 | 0.382 |
| Ad | 13 | comp257585_c0 | EC:1.1.1.284 1.1.1.1 | Alcohol dehydrogenase class-3 | -3.139 | 5.435 | 42.889 | 0.000 | 0.005 |
|  | 13 | comp261049_c0 | EC:1.1.1.35 | Hydroxyacyl-coenzyme A dehydrogenase, mitochondrial | -1.800 | 7.004 | 13.599 | 0.013 | 0.073 |
|  | 13 | comp271267_c0 | EC:6.2.1.3 | Long-chain-fatty-acid--CoA ligase 4 | -2.476 | 5.947 | 24.096 | 0.001 | 0.020 |
|  | 13 | comp271450_c0 | EC:1.3.8.6 | Glutaryl-CoA dehydrogenase, mitochondrial | -2.155 | 6.150 | 21.442 | 0.004 | 0.038 |
|  | 13 | comp273007_c0 | EC:6.2.1.3 | Long-chain-fatty-acid--CoA ligase ACSBG2 | -2.514 | 5.678 | 28.406 | 0.001 | 0.019 |
|  | 13 | comp273172_c0 | EC:5.3.3.8 | Chromodomain Y-like protein | -2.405 | 3.747 | 7.249 | 0.020 | 0.091 |
|  | 13 | comp273523_c0 | EC:1.1.1.284 1.1.1.1 | Alcohol dehydrogenase class-3 | 1.567 | 7.510 | 16.959 | 0.030 | 0.113 |
| Ad | 20 | comp257585_c0 | EC:1.1.1.284 1.1.1.1 | S-(hydroxymethyl)glutathione dehydrogenase | -3.131 | 5.435 | 41.906 | 0.000 | 0.006 |
|  | 20 | comp262350_c0 | EC:1.1.1.284 1.1.1.1 | Alcohol dehydrogenase class-3 chain L | -4.919 | 2.101 | 10.611 | 0.007 | 0.058 |
|  | 20 | comp271267_c0 | EC:6.2.1.3 | Long-chain-fatty-acid--CoA ligase 4 | -1.541 | 5.947 | 9.925 | 0.038 | 0.143 |
|  | 20 | comp271450_c0 | EC:1.3.8.6 | Glutaryl-CoA dehydrogenase, mitochondrial | -2.522 | 6.150 | 28.099 | 0.001 | 0.019 |
|  | 20 | comp273007_c0 | EC:6.2.1.3 | Long-chain-fatty-acid--CoA ligase ACSBG2 | -2.373 | 5.678 | 25.201 | 0.002 | 0.029 |

**Table S5**. Differential expression of master regulator genes (SREBP and TAp63) identified in *Calanus* C5s and adults sampled at all time points within the experiment.

|  | **SREBP** | | | | | **TAp63** | | | | |
| --- | --- | --- | --- | --- | --- | --- | --- | --- | --- | --- |
| **comparison** | **logFC** | **logCPM** | **F** | **P** | **FDR** | **logFC** | **logCPM** | **F** | **P** | **FDR** |
| C5_Day0vsRef | -0.694 | 4.559 | 3.078 | 0.320 | 0.648 | -0.801 | 2.719 | 1.779 | 0.338 | 0.660 |
| C5_Day5vsRef | -0.431 | 4.559 | 0.970 | 0.577 | 0.854 | 0.091 | 2.719 | 0.019 | 0.920 | 0.991 |
| C5_Day13vsRef | -0.793 | 4.559 | 3.114 | 0.317 | 0.607 | -0.786 | 2.719 | 1.293 | 0.414 | 0.682 |
| C5_Day20vsRef | -1.870 | 4.559 | 9.006 | 0.089 | 0.501 | -1.491 | 2.719 | 2.271 | 0.279 | 0.676 |
| Ad_Day13vsRef | -2.238 | 4.559 | 23.232 | **0.006** | 0.049 | -2.222 | 2.719 | 9.685 | **0.025** | 0.104 |
| Ad_Day20vsRef | -2.173 | 4.559 | 21.425 | **0.009** | 0.065 | -2.943 | 2.719 | 14.016 | **0.007** | 0.058 |
| C5_Day5vsDay0 | 0.263 | 4.559 | 0.437 | 0.708 | 1.000 | 0.892 | 2.719 | 2.248 | 0.282 | 1.000 |
| C5_Day13vsDay0 | -0.099 | 4.559 | 0.058 | 0.892 | 1.000 | 0.015 | 2.719 | 0.001 | 0.986 | 1.000 |
| C5_Day20vsDay0 | -1.176 | 4.559 | 4.028 | 0.256 | 0.842 | -0.690 | 2.719 | 0.530 | 0.601 | 0.878 |
| C5_Day13vsDay5 | -0.362 | 4.559 | 0.654 | 0.647 | 1.000 | -0.877 | 2.719 | 1.627 | 0.360 | 1.000 |
| C5_Day20vsDay5 | -1.439 | 4.559 | 5.512 | 0.184 | 0.840 | -1.582 | 2.719 | 2.564 | 0.250 | 0.840 |
| C5_Day20vsDay13 | -1.077 | 4.559 | 3.097 | 0.319 | 0.848 | -0.705 | 2.719 | 0.498 | 0.612 | 0.892 |
| Ad_Day13vsC5_Day0 | -1.544 | 4.559 | 12.874 | **0.042** | 0.221 | -1.421 | 2.719 | 4.535 | 0.126 | 0.335 |
| Ad_Day20vsC5_Day0 | -1.479 | 4.559 | 11.507 | 0.055 | 0.260 | -2.142 | 2.719 | 8.210 | **0.040** | 0.229 |
| Ad_Day13vsC5_Day5 | -1.807 | 4.559 | 15.532 | **0.026** | 0.193 | -2.313 | 2.719 | 10.567 | **0.020** | 0.182 |
| Ad_Day20vsC5_Day5 | -1.742 | 4.559 | 14.083 | **0.034** | 0.230 | -3.034 | 2.719 | 14.995 | **0.005** | 0.147 |
| Ad_Day20vsC5_Day13 | -1.380 | 4.559 | 8.634 | 0.096 | 0.556 | -2.156 | 2.719 | 7.209 | 0.054 | 0.529 |
| AdvsC5_Day13 | -1.445 | 4.559 | 9.717 | 0.077 | 0.472 | -1.436 | 2.719 | 3.868 | 0.158 | 0.538 |
| AdvsC5_Day20 | -0.303 | 4.559 | 0.256 | 0.774 | 0.943 | -1.452 | 2.719 | 1.862 | 0.327 | 0.676 |

**Table S5** cont. Differential expression of master regulator genes (HNF4 and NHR-E75) identified in *Calanus* C5s and adults sampled at all time points within the experiment.

|  | **HNF4** | | | | | **NHR-E75** | | | | |
| --- | --- | --- | --- | --- | --- | --- | --- | --- | --- | --- |
| **comparison** | **logFC** | **logCPM** | **F** | **P** | **FDR** | **logFC** | **logCPM** | **F** | **P** | **FDR** |
| C5_Day0vsRef | -0.823 | 4.602 | 4.151 | 0.236 | 0.574 | 0.538 | 7.784 | 1.964 | 0.410 | 0.715 |
| C5_Day5vsRef | -0.911 | 4.602 | 4.084 | 0.240 | 0.631 | 0.626 | 7.784 | 2.268 | 0.376 | 0.735 |
| C5_Day13vsRef | -1.144 | 4.602 | 6.073 | 0.152 | 0.445 | 0.165 | 7.784 | 0.159 | 0.815 | 0.939 |
| C5_Day20vsRef | 0.009 | 4.602 | 0.000 | 0.993 | 1.000 | 0.615 | 7.784 | 1.520 | 0.468 | 0.788 |
| Ad_Day13vsRef | -2.450 | 4.602 | 26.106 | **0.003** | 0.033 | -1.653 | 7.784 | 15.025 | **0.023** | 0.097 |
| Ad_Day20vsRef | -2.098 | 4.602 | 19.392 | **0.010** | 0.072 | -1.230 | 7.784 | 8.484 | 0.087 | 0.224 |
| C5_Day5vsDay0 | -0.088 | 4.602 | 0.046 | 0.901 | 1.000 | 0.088 | 7.784 | 0.055 | 0.891 | 1.000 |
| C5_Day13vsDay0 | -0.320 | 4.602 | 0.566 | 0.662 | 1.000 | -0.372 | 7.784 | 0.949 | 0.567 | 1.000 |
| C5_Day20vsDay0 | 0.832 | 4.602 | 2.673 | 0.342 | 0.846 | 0.077 | 7.784 | 0.026 | 0.924 | 0.993 |
| C5_Day13vsDay5 | -0.233 | 4.602 | 0.255 | 0.769 | 1.000 | -0.460 | 7.784 | 1.229 | 0.514 | 1.000 |
| C5_Day20vsDay5 | 0.920 | 4.602 | 2.848 | 0.327 | 0.844 | -0.011 | 7.784 | 0.000 | 0.990 | 1.000 |
| C5_Day20vsDay13 | 1.152 | 4.602 | 4.341 | 0.226 | 0.845 | 0.450 | 7.784 | 0.803 | 0.598 | 0.888 |
| Ad_Day13vsC5_Day0 | -1.627 | 4.602 | 13.462 | **0.033** | 0.201 | -2.191 | 7.784 | 28.133 | **0.002** | 0.077 |
| Ad_Day20vsC5_Day0 | -1.275 | 4.602 | 8.390 | 0.092 | 0.316 | -1.767 | 7.784 | 19.051 | **0.010** | 0.142 |
| Ad_Day13vsC5_Day5 | -1.539 | 4.602 | 10.805 | 0.056 | 0.243 | -2.279 | 7.784 | 27.456 | **0.002** | 0.107 |
| Ad_Day20vsC5_Day5 | -1.187 | 4.602 | 6.453 | 0.140 | 0.366 | -1.856 | 7.784 | 18.712 | **0.011** | 0.169 |
| Ad_Day20vsC5_Day13 | -0.955 | 4.602 | 4.007 | 0.245 | 0.646 | -1.395 | 7.784 | 10.816 | 0.053 | 0.528 |
| AdvsC5_Day13 | -1.306 | 4.602 | 7.506 | 0.111 | 0.498 | -1.819 | 7.784 | 17.972 | **0.013** | 0.415 |
| AdvsC5_Day20 | -2.107 | 4.602 | 14.633 | **0.026** | 0.318 | -1.845 | 7.784 | 14.103 | **0.027** | 0.321 |

**Table S6**. Top hit sequences and differential expression of proteasome subunits identified in *Calanus* C5s and adults sampled at all time points within the experiment. Sequences were obtained from *Drosophila melanogaster.*

| **proteasome subunit** | **C5_day 0** | **C5_day 5** | **C5_day 13** | **C5_day 20** | **AD_day 13** | **AD_day 20** |
| --- | --- | --- | --- | --- | --- | --- |
| α1 |  |  |  |  |  |  |
| α2 |  |  |  |  |  |  |
| α3 |  |  |  |  | 1.901 | 1.886 |
| α4 |  |  |  |  |  |  |
| α5 |  |  |  |  |  |  |
| α6 |  |  |  |  |  |  |
| α7 |  |  |  |  | 0.468 |  |
| β1 | 1.702 |  |  |  | 1.455 |  |
| β2 | 1.388 |  |  |  | 1.581 | 1.832 |
| β3 |  | 1.451 |  |  | 1.462 |  |
| β4 | 1.649 | 1.643 |  |  | 1.980 | 1.755 |
| β5 |  |  |  |  |  |  |
| β6 |  |  |  |  |  |  |
| β7 |  | 1.528 |  |  | 1.996 | 1.540 |

**Table S7.** Number of reads per sample. NCBI accession numbers assigned to each sample library. Ad: adults. ref: reference group. M seqs: number of reads per sample library in millions.

| **Accession no.** | **Stage** | **Time** | **Replicate** | **M seqs** |
| --- | --- | --- | --- | --- |
| SRX3459593 | C5 | ref | 1 | 7.7 |
| SRX3459594 | C5 | ref | 2 | 11 |
| SRX3459596 | C5 | 0 | 1 | 10.3 |
| SRX3459589 | C5 | 0 | 2 | 9.4 |
| SRX3459595 | C5 | 0 | 3 | 7.1 |
| SRX3459591 | C5 | 5 | 1 | 12 |
| SRX3459590 | C5 | 5 | 2 | 8.3 |
| SRX3459592 | C5 | 13 | 1 | 12 |
| SRX3459587 | C5 | 13 | 2 | 9.4 |
| SRX3459588 | C5 | 20 | 1 | 5.6 |
| SRX3459600 | Ad | 13 | 1 | 8.6 |
| SRX3459599 | Ad | 13 | 2 | 14.9 |
| SRX3459598 | Ad | 20 | 1 | 7.6 |
| SRX3459597 | Ad | 20 | 2 | 5.1 |

**Table S8a.** NCBI accession numbers for amino acid sequences encoding carnitine palmitoyltransferase 1 (CPT1) and 2 (CPT2), retrieved from the KEGG database.

| **Species** | **CPT1 accession no. (amino acid)** | **CPT2 accession no. (amino acid)** |
| --- | --- | --- |
| *Daphnia pulex* | EFX73638 | EFX87483 |
| *Drosophila melanogaster* | NP_001163111 | NP_647756 |
| *Tribolium castaneum* | XP_008191462 | XP_971442 |
| *Homo sapiens* | NP_001868  NP_001138607  NP_001186681 | NP_000089 |
| *Mus musculus* | NP_038523  NP_034078  NP_001239399 | NP_034079 |

**Table S8b**. NCBI accession number for nucleotide sequences encoding carnitine palmitoyltransferase 1 (CPT1) and 2 (CPT2), based on *Daphnia pulex* sequences, from transcriptomic databases of copepod species.

| **Species** | **CPT1 accession no.** | **CPT2 accession no.** |
| --- | --- | --- |
| *Calanus finmarchicus* | GBFB01168033.1*  GAXK01205423.1 | Not found  Not found |
| *Calanus glacialis* | Not found  HACJ01014180.1 | Not found  Not found |
| *Eurytemora affinis* | GBGO01063576.1 | Not found |
| *Tigriopus japonicus* | GCHA01034916.1 | GCHA01014159.1 |

* referred to as “comp272598_c0_seq5” in the text, which is also a unique identifier of this sequence within the NCBI BioProject.

**Table S9.** Predicted open reading frames for copepod CPT sequences.

| > GBFB01168033.1 \|ORF1 length 676 aa, 2031 bp, from 2..2032 of > GBFB01168033.1 TSA: *Calanus finmarchicus* comp272598_c0_seq5 transcribed RNA sequence |
| --- |
| KSRMFVHRARIKQSMKLLGDFQFTPTNPGYGYNVHEPYINSRPISLLSKENNHQRQLLPSLAFPNTSLRHESSKAGPGQSLSLQTQLGKLPVPSLKDTLPKFLRTVRPLVNNAEYEETFEKVKDFAKEGGIGHKLQALLEERGRQTENWFSDWWLDMAYLGYRDPVIVWSSPGIVWPTQIFQDKGEMVKFAAKAIAGALDYKISVDDQTIPVEMQGGKPLDMQQYFKVFGTTRLPATPLDKQSFNPDSKHIIVIYKNNFFKVEVYGETGEQLSAEQIQTSLEEVVKMVSNMGPEVGVLTSNNRDDWTKDFALLSNNKKNKASLKEIETALFNINLDPNYEDYAANDELSKSALISLHGGGSAHAGANRWHDKTLQIFVAESGECGMTYEHSPAEGPPLMILTDHILGYIGGTVRNGSNLPAIKYNPVQQLEFVLNDALQLAISKAKENLDSLVNEVDMHVLHFKHFGKNEIKSLGFSPDSFIQTAIQLAFYRLQKEPGAHYESGGTRQFIHGRTEVIRSCSIESVEFAKAVTQSEGTSSDKFVLMKRAIQAHNSYAKLAVAGLGVDRHLQGMKQIGVENAIEPHDLFSDTGYVKSSQMRISTSQVAGSSASFLCFGPLVSDGYGCCYNPRSDDIFFPCSALTSCPATSATEFRDALEQSLLDMRLLVLENVQQSKL |
| >GAXK01205423.1\|ORF1 length 691 aa, 2076 bp, from 106..2181 of GAXK01205423.1 TSA: *Calanus* *finmarchicus* comp158174_c2_seq1 transcribed RNA sequence |
| KSPNLIRKADRSRIMLNKSRMFVHRARIKQSMKLLGDFQFTPTNPGYNVHEPYINSRPISLLSKENNHQRQLLPSLAFPNTSLRHESSKAGPGQSLSLQNQLGKLPVPSLKDTLPKFLRTVRPLVNNEEYEETFDKVKDFAKEGGIGHKLQALLEERGRQTENWFSDWWLDMAYLGYRDPVIVWSSPGIVWPTQIFQDKGEMVKFAAKAIAGALDYKISVDDQTIPVEMQGGKPLDMQQYFKVFGTTRLPATPLDKQSFNPDSKHIIVIYKNNFFKVEVYGETGEQLSAEQIQTSLEEVVKMVSNMGPEVGVLTSNNRDDWTKDFALLSNNKKNKASLKEIETALFNINLDPNYEDYAANDELSKSALISLHGGGSAHAGANRWHDKTLQIFVAESGECGMTYEHSPAEGPPLMILTDHILGYIGGTVRNGSNLPAIKYNPVQQLEFVLNDALQLAISKAKENLDSLVNEVDMHVLHFKHFGKNEIKSLGFSPDSFIQTAIQLAFYRLQKEPGAHYESGGTRQFIHGRTEVIRSCSIESVEFAKAVTQSEGTSSDKFVLMKRAIQAHNSYAKLAVAGLGVDRHLQGMKQIGVENAIEPHDLFSDVGYVKSSQMRISTSQVAGSSASFLCFGPLVSDGYGCCYNPRSDDIFFPCSALTSCPATSATEFRDALEQSLLDMRLLVLENVQQSKL |
| >GBGO01063576.1\|ORF1 length 709 aa, 2130 bp, from 2..2131 of GBGO01063576.1 TSA: *Eurytemora affinis* comp54296_c0_seq1 transcribed RNA sequence |
| TGYPNPPPLPPTPLPPYMASSPLRSPRDSPRSSPRTPRTNRAAEVRIQSKQRSLSMTEVTKSPSRSKVDLTRSPSRSKQDLQRSSPKRSLKSQPKKDEDIGPQVYVDLPKLPLPPLKQTMDQYLDNLKPILNEADLEKVKAEVEEWMKEGGVGEKVHAHLHQKREELDNWAYQYWLRDMYMDIRIPIPVNVNPGMVFPRLDFRSTDETLSWTSRMVRELCRFKERLDNHLIPQDKAATREKGQPLCMAQYFRLMTTYRRPGLSQDSQISSGVEEKEEEHILVGRRGTWYHIPVKHNGKWKSLEDIYSSLMDVWENSENSAVLALEDRVGVMSAGDRDSWGASYAVLEEDPENKKNLELVANSLFLVCLDEETQNKSDAHRSMKDMFRQMLTGAGSRYNATNRWFDKTLQLVVTGDGVCGVCYEHSASEGIVPVVILEDIIKRVGTSWKSSIAYHPSSSNFTKLSWNLTPQLKLDINKAIQVIDSLNDENDLEVFRFQEYGKDFIKSSRCSPDAWLQLSLQLTIYRLYGYIVPTYESASTRRFQLGRVDCIRASHPEGLSWCQDMMMETKTKEEKRKSFETAIKKQTKVMIENILGYGMDIPLLGLREGTKELGLWDKQGLFKDPAFDKLNQFLLSTSQVPISLDPAFMGYGAVVPDGYGVSYNPYSSSIIFCICSFYSSPLTNSRNFATQLQRSLLDMKQLFVKGDGRK |
| >HACJ01014180.1\|ORF1 length 322 aa, 966 bp, from complement(1..966) of HACJ01014180.1 TSA: *Calanus glacialis*, contig Cglac2_c14194, transcribed RNA sequence |
| LVAGYGLPWLQGSCHCLVQSRNCLAYTDISGQGEMVKFSAKAIAGALDYKISVDNQTIPVEMQSGKPLDMQQYFKVFGTTRLPATPLDKQSFNPDSKHIIVIYRNNFFKVEVYGETGEQLSAEQIQTSLEEVVKMVXXXGPEVGVLTSNNRDDWXKDFALLSNNKKNKASLKEIETALFNINLDPXYEEYXANDDLSKSALISLHGGGSAHAGANRWHDKTLQIFVAESGECGMTYEHSPAEGPPLMILTDHILGYIGGTVRNGSNLPAIKYNPVQQLEFVLNDALHLAISKAKENLDNLVNEVDMHVLHFKHFGKNEIKYL |
| >GCHA01014159.1\|ORF1 length 662 aa, 1989 bp, from 1..1989 of GCHA01014159.1 TSA: *Tigriopus japonicus* TJ_CDS_14160 transcribed RNA sequence |
| MWSLCRTCGSIPKGVWCYSTTPYSSSQRQGAVEVKSKEYQYLQQSIVPTDKFQKSLPRLPIPKLRETCDRYLASQRPLLDQNDFTQTEKLVNQFINGPGPELDAELRRQDKDNKHTSYISGPWTDMYLKDRRPVSFTHNPGIMFAHDSRPEFMNSAWRSAHLLISTLRLLKSYQANILKPEVFHLQPTKTNTPTYWNRVKFMPNLIATPLSYMFKAFPLDMSQYDNLLQSTRIPLPQLDIIRRYPDSKHVVVLRRGHFYCFDVLDHDGNLFEPHYYLKSIQYILDHKPKGESSGIGVFTSESRDVWSEMRAHLVDHLGNEEALNKIDSGLHVICLDEWDHADEEAPKSTREIVGGSDPTNRWFDKSFSLIYSRNGAVSINFEHAWGDGVAVMRLIDEIVQDSQSNDFFAANENIHSTLHVEEIKINIDDRIKIQVGQSKKTYFEMYNSVDFEEFYFYGLGKDDCKRLQVGPDSLMQTAFQIAYHRLFGKFVPTYESCSTSIFKHGRTETVRPCTNDTKACAEAFNDSRQKDPNELLGLLQNCSKTHFEMTKNAAQGKGWDRHLFAIKDLCIRQNHPMPVLFEDPAYSKINQNVLSTSTLASHNMIHGGFCPVVPNGFGVGYQIRNDFLGVGLSTFKAHSNAKDMNDALISSFEDMKKVLKAN |
| >GCHA01034916.1\|ORF1 length 773 aa, 2322 bp, from 1..2322 of GCHA01034916.1 TSA: *Tigriopus japonicus* TJ_CDS_34918 transcribed RNA sequence |
| MAEARQAVGFQFSVTHDGNLNINFDREVLKLIYHAVERGYKKRFARLLNSLHNGIFPFGPYILIGNTLILTVLHLAGLDFFFGVTQTILQGLNFQGVLWLVSCLVSAILGSLLIVSVVRFLLRLLFSYTGWIYLSREQLRNPPFLIKVWMLIIKGLISIRLIGKESSPGHAMLRSYQGALPSLPLPSIQDTISRYLESVTPILSPEELREMKSLAEEFKASLGPRLQKYLWIKKLWSDNYVSDWWEEYVYLRGRLPLMINSNYYAVDAIFNKPTTNQSARAANVIHAAFCFRRLLQKQAPAPLMVQNFIPMCSNQYERTFNTTRVPGEVTDTLVHYDDSTHVVVAHKGRFYRVGCYHEGRLLDPAEIQNQIQGILNDKVEGDDGEKLLGVMTAANRTEWAKFRSKFCSDGANWNSLKVIDTAAFVVILDDYSYDYDPEDPSQLNNFGRKMLHGKGYDRWFDKSLCLIVGQNARIGLNAEHSWADAPVVGHFWEYVLVNDSEILGYDENGNCKGEVCTSSLSAIKLRWVFPKEAIDVMDKSLITAEAMIEDLDLYILPFFKFGKGAAKKGNLSPDAFIQLSLQLTYFRNAGQFCQTYEACMTRLFRDGRTETVRPCTNEAATWVRAMDDPNVDKKTKLDLLKLACAKHQKSYQDCMTGKGVDRHLFCLYVVSKYLEEKSPFLEKVLSEPWRLSTSQTAVRGEYLDVRKYPELISCGGGFGPVADDGYGVSYIIIGEDRIFFHVSSKKSCPITSSSRFAEMLGQSLTDVKNLFEV |

**Figure S1.** Most statistically significantly enriched GO terms (lowest P-values) in the reference group compared to copepodites from day 0, 5, 13 and 20 combined. MF: Molecular function. CC: Cell component. BP: Biological process.

**Figure S2.** Most statistically significantly enriched GO terms (lowest P-values) in the copepodites from day 0, 5, 13 and 20 combined compared to the reference group. MF: Molecular function. CC: Cell component. BP: Biological process.

**Figure S3**. Differential expression (log2 fold change) of molecular markers of diapause and development in *C. finmarchicus* copepodites sampled at day0 (T0), 5 (T1), 13 (T2) and 20 (T3) and adults from day 13 and 20, compared to the reference group. A log2 fold change of -2 signifies a 4 fold decrease from the reference group
